# Clinical Diagnosis of Primary Syphilis

**Published:** 1929

**Authors:** A. Michael Critchley


### CLINICAL DIAGNOSIS OF PRIMARY SYPHILIS.

BY

A. Michael Critchley, M.D.

---

The tendency of the modern practitioner is to rely more and more on pathological and X-ray findings, to the exclusion of clinical investigation in arriving at a diagnosis. Whilst the undoubted value of the pathologist and radiographer, as aids to diagnosis, is not disputed, it must be remembered that they are only "aids" and are not indispensable, and that clinical observations are still the key to correct diagnosis. The present-day syphilologist often tends to subordinate his own clinical findings to the frequently confusing reports of the pathologist, much to the detriment of his patients.

In order to get the best results in treating syphilis the infection must be diagnosed before the Wassermann reaction becomes positive, and treatment must be started without delay. When a patient presents himself suffering from a venereal sore the problem is to find out as quickly as possible whether or not this sore is syphilitic, and to apply immediate treatment. According to Stokes,[1] the diagnosis of syphilis is a laboratory problem, and clinical appearances are worthless. Thus Stokes when confronted with a sore employs the following routine. A dark-field examination of the sore and adjacent glands is made, and local and blood complement fixation tests are performed. If the dark-ground examinations fail to reveal spirochætes after repeated attempts, Stokes does a Wassermann follow-up ; that is, the blood is tested weekly for four weeks and monthly for four months, at the end of which period, if the blood is still negative, it is then presumed that the sore was non-syphilitic. But what a tragedy if the final Wassermann is positive — four months wasted, during which the infection has become disseminated throughout the body. This generalization could have been prevented if a clinician who had the courage of his convictions had seen the patient in the first instance. Stokes claims that 82 per cent. of chancres have demonstrable spirochætes, which is a most optimistic finding ; 42 per cent. would be a much more correct estimate. Again and again one sees a report on a scraping "No spirochætes found," only to be greeted with a second report a few weeks later, "Blood Wassermann + +." It must be emphasized that once the Wassermann reaction has become positive it is impossible to guarantee cure, irrespective of the amount of treatment given. When a syphilitic sore is treated before generalization has set in, a cure, provided the treatment is adequate, can safely be guaranteed.

The clinical features of a genital chancre vary slightly according to the exact situation of the sore, but wherever situated there are always certain findings common to all. The most important item in examination is inspection. As soon as one starts feeling the sore, mistakes become more liable. Induration, which is such a characteristic trait of primary sores, can be seen more surely than felt, for all the normal folds of the skin around the sore become obliterated owing to the swelling and induration. The sore is usually painless. When the chancre is secondarily infected, it can be seen by the way the patient manipulates his penis that it causes but little discomfort. Erosion is more common than ulceration. An ulcerative chancre has regular non-undermined edges, it does not appear to be healing in one pole and spreading in the other like an *ulcus molle*, and the base is covered by a serous non-purulent exudate. Bilateral inguinal adenitis is usual. It is a mistake to say that chancres are rarely multiple, for in about 25 per cent. of cases there is more than one primary sore. Many chancres are missed because gonorrhœa conceals the syphilitic lesion ; especially is this mistake made in meatal sores, and in patients being treated for gonorrhœa who subsequently develop syphilis which they acquired at the same time as the urethritis. The genitalia of each venereal patient should be examined every time he attends for treatment.

The easiest sores to diagnose are erosive chancres occurring on the inner surface of the prepuce and on the corona most commonly near the frænum. In these cases, owing to the induration the foreskin rolls back in an absolutely pathognomonic manner in one big piece, and the normal folds of mucous membrane are entirely obliterated.

Sometimes a chancre becomes secondarily infected, and phagedænic ulceration may result. In this type of sore a search for spirochætes is waste of time, and until the Wassermann becomes positive the diagnosis must be made solely on clinical observation. Severe pain is absent, and there is comparatively little inflammatory reaction of the skin. If the phagedæna is due to a soft sore, which is a rare occurrence compared with syphilitic phagedæna, the skin reaction is marked. A bubo in the groin in these secondarily infected ulcers is no contra-indication to a diagnosis of syphilis, for there is no reason why some of the secondary organisms should not travel along the lymphatics to the inguinal glands and there provoke suppuration.

Intra-urethral chancres are another source of difficulty, but fortunately it is extremely uncommon for a sore to be entirely intra-urethral, as usually one pole of the sore is external to the meatus. Again, the absence of the normal folds of mucous membrane is to be observed. Digital examination if done carelessly will miss any induration, for as a rule it can only be felt in the long axis of the urethra and not from side to side.

Sometimes a thorough examination of a sore cannot be made owing to the presence of phimosis. These cases can still be diagnosed clinically without having to resort to surgical measures such as dorsal slit. If the sore is a chancre there is a definite bulging of the prepuce on that side, but there is neither reddening nor tenderness of the foreskin. On palpation induration may be felt.

Differential diagnosis of chancre includes the consideration of such lesions as *ulcus molle, herpes preputialis* and scabies. Provided each sore is examined carefully there should be a little difficulty in arriving at the correct diagnosis. Pathological examinations in these cases are useful if positive, but if negative they simply confuse and introduce an element of doubt.

REFERENCE.

[1] Stokes, J. H., *Modern Clinical Syphilology,* 1926.

